# Domino liver transplantation for select metabolic disorders: Expanding the living donor pool

**DOI:** 10.1002/jmd2.12053

**Published:** 2019-06-19

**Authors:** Neslihan Celik, James E. Squires, Kyle Soltys, Jerry Vockley, Diana A. Shellmer, Wonbae Chang, Kevin Strauss, Patrick McKiernan, Armando Ganoza, Rakesh Sindhi, Geoffrey Bond, George Mazariegos, Ajai Khanna

**Affiliations:** ^1^ Hillman Center for Pediatric Transplantation, Children's Hospital of Pittsburgh of UPMC Thomas E. Starzl Transplantation Institute, University of Pittsburgh School of Medicine Pittsburgh Pennsylvania; ^2^ Pediatric Hepatology Children's Hospital of Pittsburgh of UPMC Pittsburgh Pennsylvania; ^3^ Division of Medical Genetics University of Pittsburgh School of Medicine, Center for Rare Disease Therapy, Children's Hospital of Pittsburgh of UPMC Pittsburgh Pennsylvania

**Keywords:** domino liver transplantation, live liver donation, maple syrup urine disease, metabolic liver disease, pediatric transplantation

## Abstract

Domino liver transplantation (DLT) involves transplanting liver from a patient with metabolic disease into a patient with end‐stage liver disease with the expectation that the recipient will not develop the metabolic syndrome or the recurrent syndrome will have minimal affect. The domino donor gets a deceased donor or a segment of live‐donor liver through the deceased donor organ allocation system. Waitlist mortality for the domino recipient exceeds morbidity associated with getting the donor disease. Between 2015 and 2017, four patients with three metabolic disorders at UPMC Children's Hospital of Pittsburgh underwent DLT with domino allografts from maple syrup urine disease (MSUD) patients. These included patients with propionic acidemia (PA) (n = 1), Crigler‐Najjar (CN) syndrome type‐1 (n = 2), and carbamoyl phosphate synthetase deficiency (CPSD) (n = 1). Mean follow‐up was 1.6 years (range 1.1‐2.1 years). Total bilirubin levels normalized postoperatively in both CN patients and they maintain normal allograft function. The PA patient had normal to minimal elevations of isoleucine and leucine, and no other abnormalities on low protein diet supplemented with a low methionine and valine free formula. No metabolic crises have occurred. The patient with CPSD takes normal baby food. No elevation in ammonia levels have been observed in any of the patients. DLT for a select group of metabolic diseases alleviated the recipients of their metabolic defect with minimal evidence of transferrable‐branched chain amino acid elevations or clinical MSUD despite increased protein intake. DLT using allografts with MSUD expands the live donor liver pool and should be considered for select metabolic diseases that may have a different enzymatic deficiency.

**Abbreviations**
ATP: adenosine triphosphate
BCAA: branched chain amino acid
BCKDH: branched chain keto‐acid dehydrogenase enzyme complex
CMV: cytomegalovirus
CNS: Crigler‐Najjar syndrome
CPSD: carbamoyl phosphate synthetase deficiency
DLT: domino liver transplantation
EBV: Epstein‐Barr Virus
GT: gastrostomy tube
MMF: mycophenolate mofetil
MSUD: maple syrup urine disease
PA: propionic acidemia
POD: postoperative day
TCMR: T‐cell mediated rejection

## 1 INTRODUCTION

Monogenic diseases are a group of disorders caused by inheritance of single‐gene defects. Depending on the specific metabolic deficiency, native livers may exhibit significant parenchymal damage or may be structurally normal. In select disorders, liver transplantation can function as a surgical gene replacement therapy. Furthermore, certain monogenic diseases, such as MSUD, have enabled pioneering of DLT. DLT has been performed where a single‐enzyme is deficient, and the liver is morphologically normal. These explanted livers are utilized as allografts to expand the donor pool.^1^, ^2^ These procedures are pursued with the prospect that DLT recipients will not develop clinical or subclinical manifestation of enzyme defect associated with the donor liver. However, there are several reports that suggest DLT allografts can transmit the primary disease in the post‐DLT period.^3^, ^4^, ^5^, ^6^, ^7^, ^8^, ^9^, ^10^ Several DLT have been performed using livers from MSUD patients. To date there have been no reported cases of de novo clinical disease transmission associated with transplanting MSUD livers. This is due to the established observation and report by Khanna et al that branched‐chain alpha‐keto dehydrogenase complex made by the extrahepatic tissues in the domino recipient is sufficient to metabolize leucine, isoleucine and valine; the amino acids responsible for MSUD syndrome.^2^

**Figure 1:**
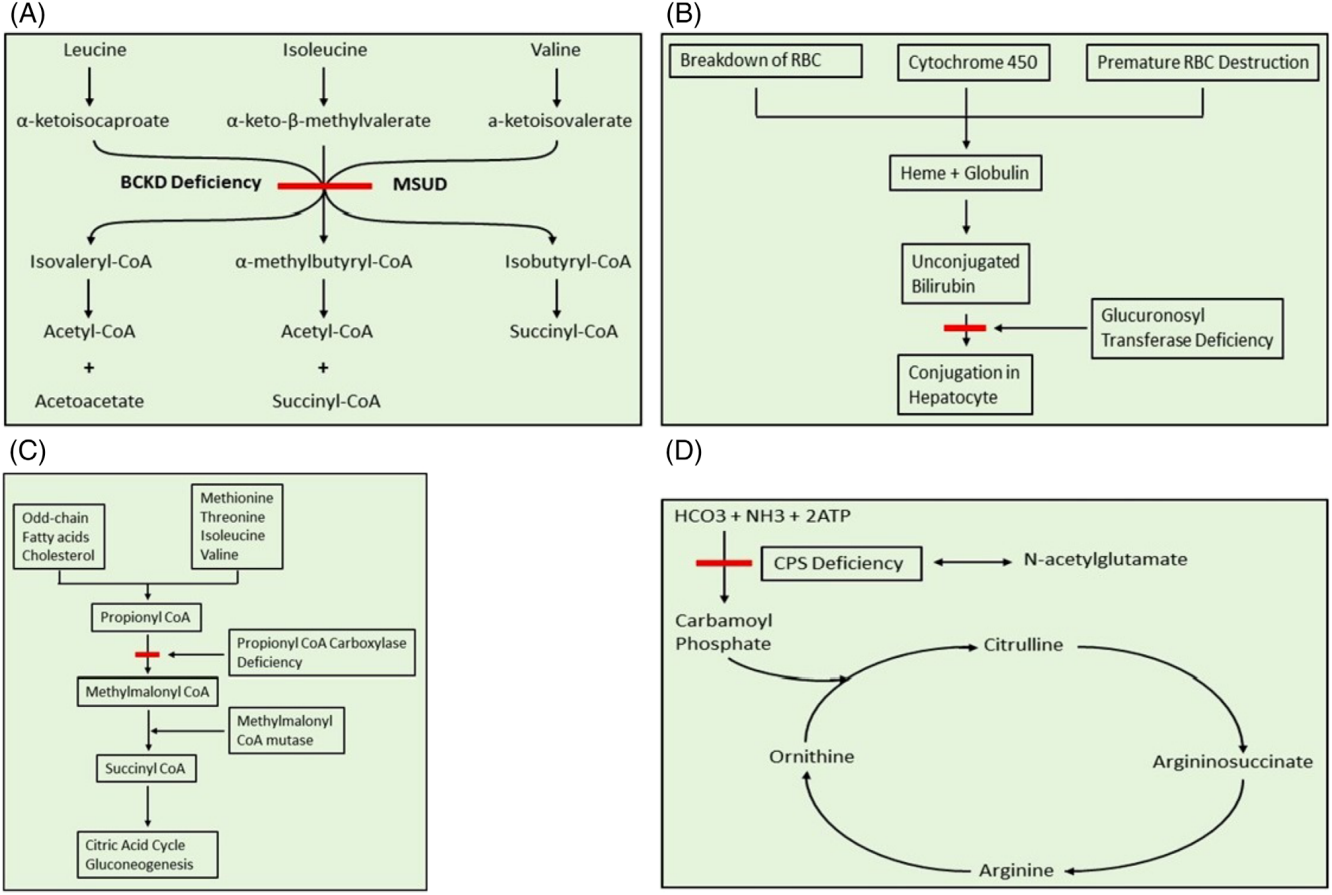
A, Maple syrup urine disease (MSUD). BCKD, branched‐chain α‐keto acid dehydrogenase. B, Bilirubin uridine disphosphonate glucuronosyl transferase deficiency in Crigler‐Najjar syndrome. C, Propionyl CoA carboxylase deficiency in propionic acidemia. D, Carbamoyl phosphate synthetase (CPS) deficiency

MSUD is an organic acidemia caused by deficiency of branched chain keto‐acid dehydrogenase enzyme complex (BCKDH). Despite optimal nutritional management, neurotoxic accumulation of branched chain amino acids derivatives (especially leucine) can occur leading to episodes of life‐threatening elevated leucine levels with cerebral edema, brain damage, and even death (Figure 1A). Symptomatic correction of MSUD is achievable with liver transplantation. While liver is only responsible for approximately 9 to 13% of BCKDH activity in whole body, enzymatically normal deceased or live‐donor liver allografts (including from carrier parents) have been shown to be sufficient for the degradation of toxic metabolites in MSUD recipients.^11^, ^12^, ^13^, ^14^, ^15^ Conversely, since majority of BCKDH activity is extrahepatic, MSUD livers have been shown to be ideal candidates for DLT donation.

DLT with MSUD livers has been performed successfully in a hemophiliac with HCV/HIV related cirrhosis^16^ and for pyruvate carboxylase deficiency.^17^ The latter is the only reported case of successful DLT into a patient with monogenic disease and normal hepatic parenchyma. We present our experience with DLT from MSUD patients for specific metabolic diseases to replace the deficient enzyme through DLT.

## 2 METHODS

A retrospective review was performed at Children's Hospital of Pittsburgh of The University of Pittsburgh Medical Center on all metabolic disease patients with a specific enzyme deficiency, normal hepatic parenchyma and preserved function who received DLT from MSUD. Medical records were analyzed, and data collected relating to patient and graft characteristics, posttransplantation complications, metabolic outcomes, patient and graft survivals. This study was approved by the institutional review board.

## 3 RESULTS

Between 2015 and November 2018, four children with three monogenic liver diseases and normal hepatocellular architecture underwent DLT with allografts from MSUD patients. These included propionic acidemia (PA, n = 1), Crigler‐Najjar syndrome type‐1 (CN‐1, n = 2) and carbamoyl phosphate synthetase deficiency (CPSD, n = 1). Mean follow‐up time after DLT was 1.6 years (range 1.1‐2.1 years).

### 3.1 Propionic acidemia

Propionic acidemia is an autosomal recessive metabolic disorder resulting from propionyl‐CoA carboxylase deficiency. It presents in the early neonatal period with poor feeding, vomiting, lethargy, and hyperammonemia. Patients can rapidly deteriorate with cardiomyopathy, infections or stroke due to buildup of propionyl CoA and propionic acid (Figure 1C).

An 11‐year‐old girl presented with ketoacidotic crisis and neutropenia related to propionic acidemia. Her medical history was notable for frequent episodes of metabolic decompensation with hospitalization every 2 to 5 months triggered by illness, central nervous system complications (including moderate intellectual disability, seizures, and hypotonia), feeding difficulties requiring gastrostomy tube (GT) feeds, sensorineural hearing loss, asthma, and femoral deep vein thrombosis. Outpatient management had consisted of 76% restricted‐24% complete protein in a 1244 cal/day diet (40 kg nutritional calculation weight). Despite optimal nutritional management, adequate metabolic control remained elusive.

Given her history of frequent metabolic decompensation and ketoacidotic crises, she was evaluated and deemed to be a good candidate for liver transplantation. The patient received a whole organ domino allograft from a MSUD donor in November 2015. Induction immunosuppression included methylprednisolone, tacrolimus and steroid cycle. The operative procedure was complicated by hepatic artery thrombosis, necessitating reconstruction with an iliac arterial interposition graft between recipient proper hepatic artery and allograft common hepatic artery. Posttransplant pleural effusion was treated with pleurocentesis. Epstein‐Barr virus (EBV) hepatitis (posttransplant 2nd month) was treated by lowering immunosuppression and a single dose of Rituximab (Genentech), T‐cell mediated rejection (TCMR posttransplant 9th month) was treated with methylprednisolone bolus, steroid recycle and mycophenolate mofetil (MMF) was added to the regimen to lower long‐term tacrolimus exposure. She received total parenteral nutrition with 1.5 to 2 g/kg protein and then switched to a more liberalized protein diet and GT feeds. At postoperative 4th month, she developed biliary stricture and was successfully managed with percutaneous transhepatic cholangiography, biliary dilation, and stent placement. Stent was subsequently removed after 4 weeks. She remains on Tacrolimus and MMF immunosuppression, and at the last follow up had normal to minimal elevations of isoleucine and leucine (Table 1), and no other amino acid abnormalities. Acyl carnitine profile continued to be consistent with her primary diagnosis. C3 metabolites were not evaluated pretransplant. Posttransplant C3 mean was 65.5 μMol/L (54.7‐119.5) (normal <0.77). Free carnitine mean posttransplant was 73.2 μMol/L (47‐89) (normal 22‐65). The patient was continued on carnitine supplementation. Gas chromatography/mass spectrometry analysis of organic acids in urine derived from the patient on day one posttransplant reveals modest lactic aciduria without ketonuria, 3‐hydroxypropionate, and methylcitrate. The pattern of excretions has somewhat normalized post‐Tx as tiglyglycine and proponylglycine are no longer observed.

**Table 1:** Clinical characteristics of domino liver donors and recipients

DLT recipient	Genetic mutation	Post DLT Follow up time (months)	Age (year) and weight (kg) at time of DLT		Post‐op complications	Mean posttransplant BCAA levels (μmol/L; mean ± sd)			Mean posttransplant day for BCAA assessment (range)	Current nutritional status	Current status, ALT/ GGT (IU/L)
			Non‐MSUD recipient	MSUD donor		Isoleucine	Leucine	Valine			
PA	PCCA p. G117D homozygous	36	11.7/39	5.1/21	Hepatic artery thrombosis, TCMR, EBV hepatitis	68 ± 40	101 ± 56	155 ± 73	94 (1‐272)	Regular diet with Propimex‐2	Alive, 42/34
CN‐1	UGT1A1 c.222c>a (p. y74^*^)	34	13.8/64	29.1/68	TCMR	85 ± 25	126 ± 33	217 ± 71	175 (7‐342)	Regular diet	Alive, 25/31
CN‐1	NA	27	20.7/57	18/69	TCMR	64	110	198	450	Regular diet	Alive, 12/7
CPSD‐1	CPS1 c.306_311 dup and GAA TGG and c.4022 del G	23	1.4/11, received left lateral segment graft	27.1/80	TCMR Steatosis, CMV hepatitis	34 ± 15	50 ± 14	89.5 ± 21	7 (1‐16)	Regular diet	Alive, 55/15

Urine analysis on postoperative day 6 revealed 3‐hydroxypropionate, propionylglycine, methylcitrate, and tiglylglycine. A modest excretion of 2‐methylbutylglycine was observed. Lactate and ketones were normal. No indication of additional branched chain amino acid analytes was observed. The analytes specific to PA had moderated since transplant. Posttransplant urine analysis, therefore, did show persistent, though improved excretion of PA‐specific analytes and no branch chain amino acids.

Postoperative mean arginine and glutamine levels were 03.2 μmol/L (normal 30‐150) and 573.0 μmol/L (normal 320‐870).

Plasma AA profiles posttransplant day 6 showed elevated glycine levels that remained below patient's historic levels. A modest elevation of alanine was observed however, no allo isoleucine was detected.

Plasma amino acid profiles normalized 2 weeks following liver transplant, however glycine and alanine remained elevated. The patient consumes regular age‐appropriate 1479 Cal/day diet consisting of 70% intact protein.

At last follow up, the patient was taking a regular age‐appropriate diet (1479 Cal/day). She continued to receive amino‐acid modified supplementation (Propimex, Abbott laboratories) and her total protein intake was 1.5 g/kg/day, 75% of which was intact protein.

No metabolic crises have occurred during the 20.1 months follow up period. She continues to grow and has not shown any deterioration in neurological status.

### 3.2 Crigler‐Najjar type 1 syndrome (CN‐1)

Crigler‐Najjar syndrome is an autosomal recessive genetic disorder in which patients are unable to convert unconjugated bilirubin to conjugated bilirubin due to deficiency of the enzyme glucoronyl transferase in hepatocytes (Figure 1B). Infants develop severe unconjugated hyperbilirubinemia in the postpartum period followed by kernicterus and encephalopathy.

A 13‐year‐old girl presented with a total bilirubin range of 11 to 14.8 mg/dL under 9 to 11 hours/day phototherapy for CN‐1. She also suffered from pruritus and nausea. She received a whole domino allograft from an MSUD donor in December 2015. Her pretransplant total bilirubin of 14.2 mg/dL normalized by 11 days posttransplant. She had an episode of TCMR at POD10 which was responsive to steroid treatment and addition of MMF. Her posttransplant amino‐acid profile was normal. She is currently receiving Tacrolimus and MMF immunosuppression and is on an unrestricted diet.

A 20‐year‐old female with CN‐1 who received 8 to 10 hours/day of phototherapy presented with a total pretransplant bilirubin range 17.4 to 19.4 mg/dL. She received a whole‐organ domino allograft from an MSUD donor in August 2016. Her total bilirubin normalized in 12 days. She had one episode of TCMR on POD13 which was unresponsive to steroid treatment and required rabbit antithymocyte globulin. Her posttransplant amino‐acid profile was normal. She continues tacrolimus and azathioprine immunosuppression and is asymptomatic on an unrestricted diet.

### 3.3 Carbamoylphosphate synthetase 1 deficiency (CPSD‐1)

Carbamoylphosphate synthetase 1 deficiency is an autosomal recessive disorder of the urea cycle. Absence of the enzyme in mitochondria blocks the first step of the urea cycle in which ammonia, ATP and bicarbonate combine to form carbamoyl phosphate that enters the urea cycle (Figure 1D). Absence of this enzyme results in hyperammonemia, protein intolerance, ataxia, seizures, and progressive mental retardation.

A 10‐month‐old girl presented with a history of CPSD‐1 with frequent metabolic disturbances requiring hospitalizations due to metabolic crises with hyperammonemia, hypercalcemia‐nephrocalcinosis, and cardiac dysfunction. This constellation of presentation in CPSD‐1 has not been reported before. The patient was managed with a low protein diet (1.2 g/kg/day) consisting of 82% restricted‐18% complete protein mostly via GT and had ammonia levels ranging between 50 and 70 μmol/L when not in crisis. Due to the severity of urea cycle defect and to improve long‐term survival and preserve neurologic functions and developmental skills, she was listed for liver transplantation. She received a left lateral segment allograft domino live‐donor liver transplantation at age 16 months from a 27‐year‐old MSUD patient in November 2016. Postoperatively patient had TCMR, which resolved with steroid treatment and addition of MMF. A liver biopsy performed for elevated transaminases on POD 24 showed diffuse steatosis. This finding totally normalized following biopsies at 4 months posttransplant with proper nutrition and vitamin E supplement. Cytomegalovirus (CMV) hepatitis at POD 48 was treated successfully with 14 days of IV Ganciclovir and CMV specific immunoglobulin. She was allowed a regular diet post‐LT and received Similac PM 60/40 (Abbott) infant formula. Leucine, isoleucine, and valine levels have been normal and no elevation in ammonia has been observed. Patient characteristics, postoperative complications and postoperative branched‐chain amino acid (BCAA) levels are shown in Table 1. She is doing well on an unrestricted diet.

## 4 DISCUSSION

DLT with allografts from patients with various monogenic diseases have been performed to address organ shortage and expand the live donor pool for liver transplantation. Furtado et al performed the first DLT with livers from patients with familial amyloidotic polyneuropathy for liver cancer patients with a short life expectancy.^1^ Tzakis et al emergently transplanted the liver from a neurogenic intestinal pseudo‐obstruction patient receiving a multivisceral organ transplantation into a patient with acute graft failure due to hepatic artery thrombosis.^18^ Between 1998 and 2017, the Domino Liver Transplant Registry has reported a total of 1254 domino transplants from 66 centers in 21 countries.^19^ The domino donor livers have mostly come from familial amyloidotic polyneuropathy patients, with occurrence of this disorder in the recipient as a prime concern. In the series of DLT from patients with familial amyloidotic polyneuropathy by Azoulay et al^10^ DLT recipients showed elevated levels of TTR‐met 30. The levels plateaued 3 to 4 weeks after DLT. Nine of the 10 reported patients underwent neurological evaluation and electrophysiologic studies. None had evidence of autonomic dysfunction. Similar concerns exist with DLT with livers form patients with primary hyperoxaluria, acute intermittent porphyria, and familial homozygous hypercholesterolemia since the liver is the main source of the production of the abnormal metabolites in these metabolic disorders.^3^, ^4^, ^5^, ^6^, ^7^, ^8^, ^9^, ^10^, ^11^, ^12^

In contrast, recurrence of de novo disease has not been observed in MSUD DLT recipients, since the liver accounts for a minority of whole‐body BCKDH activity.^2^ Recent reports have established MSUD DLT as a safe, long‐term transplant intervention, rather than as a temporary bridge.^19^, ^20^, ^21^, ^22^, ^23^ We have previously reported a series with 100% patient and graft survival and no metabolic derangement following DLT using MSUD donors with short‐term follow‐up.^12^ The literature and this series report recipients with normal plasma amino acid profiles and no metabolic decompensations following transplantation, confirming that sufficient extrahepatic BCKDH activity enables unrestricted protein intake with no clinical signs of MSUD.^2^, ^17^, ^20^, ^21^, ^22^

MSUD DLT has been performed in patients with various enzyme deficiencies. Matsunami et al reported successful short‐term results from transplanting MSUD liver to a patient with pyruvate carboxylase deficiency,^17^ and this study confirms normal metabolic balance >1‐year posttransplant. Additional case reports using domino auxiliary partial orthotopic liver transplantation technique have been described.^24^, ^25^ In the current series, CN‐1 patients achieved dramatic correction of their hyperbilirubinemia with uneventful posttransplant course. Significant metabolic improvement and clinical correction was also seen in our PA and CPSD‐1 patients. These results were expected given the underlying pathophysiology of these diseases. An MSUD liver provides normal ureagenesis for the patient with CPSD‐1, and thus provides a complete metabolic correction. The situation is more complicated in the PA patient. In this setting, a block in flux from isoleucine and valine to propionyl‐CoA in an MSUD liver would, in theory, reduce the overall propionate load to the body compared to use of a metabolically normal liver, thus providing a potential advantage to the PA recipient. However, additional whole body amino acid flux studies would be necessary to prove this hypothesis.

The technical aspects of DLT are reported in a large adult series and in limited pediatric cases, verifying feasibility and safety of the procedure without adding additional risk for donors and recipients. Younger donor age, shorter cold ischemia time, less intraoperative transfusion requirement, less graft dysfunction, and less ischemia preservation injury have been reported in DLT transplants.^19^, ^20^, ^21^, ^22^ The technical issue of hepatic artery thrombosis in our patient may be related to pathophysiology of the underlying PA, as vascular thrombosis incidence has been reported to be increased in this population.^26^

Successful DLT from a patient with one metabolic disease donor to one with a different metabolic disorder is a very complex clinical scenario necessitating a well‐organized, multidisciplinary team, requiring detailed metabolic evaluation, care, and monitoring. All patients currently have normal graft functions 1.6 years (range 1.1‐2.1 years) following DLT. No patient developed recurrent disease or graft failure. While the short‐term success in our series of DLT patients is encouraging, longer term neurocognitive follow‐up is critical to understanding its ultimate efficacy, since one of the main long‐term sequela in metabolic disease is neurologic damage. Long term follow‐up is also necessary to understand the impact on quality of life, including improvement in dietary management. Additional studies to assess stability of BCKDH homeostasis over time will be important.

## 5 CONCLUSION

We present our experience with DLT for select metabolic diseases demonstrating improved transplant‐indicated dysfunction with excellent patient and graft survival. DLT using allografts with specific metabolic disorders expands the donor pool to meet the requirement for pediatric liver transplantation. Domino allografts from patients with MSUD should be considered for patients with select metabolic derangements as there has been no evidence of branched‐chain amino acid elevation in recipients of MSUD livers. Ongoing studies of long‐term outcomes for DLT recipients, including neurocognitive status and quality of life, will be important to better define the risks and benefits of this procedure.

## CONFLICT OF INTEREST

The authors declare that they have no conflict of interest.

## INFORMED CONSENT

All procedures followed were in accordance with the ethical standards of the responsible committee on human experimentation (institutional and national) and with the Helsinki Declaration of 1975, as revised in 2000.^5^ Informed consent was obtained from all patients for being included in the study.

## AUTHOR CONTRIBUTIONS

N.C., J.E.S., K.S., G.M., and A.K. involved in conceptualization, planning, and writing the manuscript. J.V., K.S., P.M., and R.S. involved in conceptualization and planning the manuscript. D.A.S. involved in planning and providing social support. W.C. made the figures for the manuscript. A.G. and G.B. involved in planning the manuscript.
